# A cross-sectional survey of hepatitis B virus screening in patients who received immunosuppressive therapy for rheumatoid arthritis in Japan

**DOI:** 10.1186/s40780-024-00339-9

**Published:** 2024-04-18

**Authors:** Yuki Yanagisawa, Shungo Imai, Hayato Kizaki, Satoko Hori

**Affiliations:** Division of Drug Informatics, Keio University Faculty of Pharmacy, 1-5-30 Shibakoen, Minato-ku, Tokyo, 105-8512 Japan

**Keywords:** Hepatitis B virus screening, rheumatoid arthritis, Antirheumatic drugs, Immunosuppressive therapy

## Abstract

**Background:**

Patients with a history of hepatitis B virus (HBV) infection who are receiving immunosuppressive therapy are at risk of HBV reactivation and disease. Therefore, HBV screening is required prior to administering antirheumatic drugs with immunosuppressive effects. This study aimed to determine the status of hepatitis B surface antigen (HBsAg), hepatitis B core antibody (HBcAb), and hepatitis B surface antibody (HBsAb) screening prior to the initiation of drug therapy, including new antirheumatic drugs, in patients with rheumatoid arthritis.

**Methods:**

This retrospective cross-sectional study used data from April 2014 to August 2022 from the Japanese hospital-based administrative claims database. The inclusion criteria were rheumatoid arthritis and first prescription date of antirheumatic drugs.

**Results:**

A total of 82,282 patients with rheumatoid arthritis who were first prescribed antirheumatic drugs between April 2016 and August 2022 were included. Of the eligible patients, 9.7% (*n*=7,959) were screened for all HBV (HBsAg, HBsAb, and HbcAb) within 12 months prior to the date of initial prescription. The HBsAg test was performed in 30.0% (*n*=24,700), HBsAb test in 11.8% (*n*=9,717), and HBcAb test in 13.1% (*n*=10,824) of patients. The proportion of patients screened for HBV infection has been increasing since 2018; however, the proportion of patients screened for rheumatoid arthritis remains low.

**Conclusions:**

Our findings suggest that HBV screening may be insufficient in patients who received antirheumatic drugs. With the increasing use of new immunosuppressive antirheumatic drugs, including biological agents, healthcare providers should understand the risk of HBV reactivation and conduct appropriate screening.

**Supplementary Information:**

The online version contains supplementary material available at 10.1186/s40780-024-00339-9.

## Background

Hepatitis B virus (HBV) infects hepatocytes and leaves two strands of closed circular DNA inside the host nucleus. Therefore, HBsAg-positive carriers and previously infected patients who are negative for hepatitis B surface antigen (HBsAg) and positive for hepatitis B surface antibody (HBsAb) or hepatitis B core antibody (HBcAb) may develop severe and fatal hepatitis owing to high serum levels of HBV-DNA after immunosuppressive therapy or chemotherapy [1]. In addition, reactivation of HBV can lead to an interruption of immunosuppressive therapy and chemotherapy, thereby decreasing treatment efficacy. This complication can be prevented by HBV screening prior to immunosuppressant therapy and subsequently initiating antiviral prophylaxis in patients with chronic HBV infection.

The importance of recognising the risk of HBV reactivation has been emphasised in Japan and overseas. The American Association for the Study of Liver Diseases (AASLD) [2], European Association for the Study of the Liver (EASL) [3], and Asian Pacific Association for the Study of the Liver (APASL) [4] recommend the evaluation of HBcAb, HBsAb, and HBsAg through serological tests for preventing and monitoring HBV reactivation.

The Japan College of Rheumatology (JCR) and Japan Society of Hepatology (JSH) recommend that all patients who start immunosuppressive therapy, including biological disease-modifying antirheumatic drugs (DMARDs), should have HB antigen levels checked before starting therapy, and if positive, a hepatologist should be consulted for treatment with a nucleic acid analogue. If a patient is positive for HBsAb or HBcAb, he or she perform HBV-DNA quantification, as needed administered prophylactic nucleic acid analogue therapy [5, 6].

In Japan, 13 cases of HBV reactivation without complying HB treatment guidelines owing to immunosuppressive therapy or chemotherapy have been reported between 2017–2020, and an alert has been issued (Medical Accident Collection Project, "Medical Safety Information No. 171”, February 2021, In Japanese). A nationwide survey of acute liver failure in Japan conducted in 2011 has revealed an increase in cases of fulminant hepatitis caused by HBV reactivation owing to immunosuppressive therapy among previously HBV-infected patients [7]. HBV infection occurs worldwide, with a (HBsAg-positive) proportion of 0.7% and previous HBV infection (HBsAb or HBcAb-positive) proportion of 23% in Japanese patients suffering from rheumatoid arthritis [8], and other reports are comparable [9–11]. The proportion of HBsAg-positive patients is < 2% in North America [12] and 5–7% in Taiwan [13]. Although the proportion of HBV infection is lower in Japan than in other countries, a certain number of patients with previous HBV infection exists. Therefore, HBV screening is essential for preventing reactivation.

No prospective studies have reported the proportions of HBV reactivation in patients with rheumatoid arthritis; however, meta-analyses [14] and case reports [15] on patients receiving DMARDs have reported the proportions of HBV reactivation of 15–39% and 3–5% in patients with rheumatoid arthritis positive or negative for HBsAg, respectively, and receiving tumour necrosis factor (TNF) inhibitors. Targeted synthetic (ts)DMARDs and abatacept, a non-TNF-α drug, are a risk factor for HBV reactivation [16, 17]. In this context, HBV screening (i.e., any of HBsAb or HBcAb or HBV-DNA quantification) proportions for patients with rheumatoid arthritis are increasing, with a database-based survey in 2018 reporting proportions of 20.3% in North America and 24.5% in Taiwan prior to initiation of immunosuppressive therapy [18]. The incidence of HBV reactivation is 1.5–5% in Japanese patients with rheumatoid arthritis [19–23]. Nevertheless, a survey of HBV screening proportions in 2013–2014 using the Japanese national database have shown that the screening proportions for HBsAg, HBsAb, and HBcAb are 28.23%, 12.52%, and 14.63%, respectively, among 76,641 Japanese patients with rheumatoid arthritis [20]. Although the proportion of all these tests (HBsAg, HBsAb, and HBcAb) is not specified, it is assumed to be < 20%, which is not enough compared to those of other countries. The JCR has presented "Recommendations for Immunosuppressive Therapy for Patients with HBV-Infected Rheumatic Disease" in 2011, and the 4^th^ revised edition was published in 2014; however, the most recent HBV screening proportions in a domestic multicentre setting have not been reported after the revision.

The total number of approved Janus kinase (JAK) inhibitors is five in Japan, including tofacitinib citrate, which was approved in 2013, and baricitinib, peficitinib hydrobromide, upadacitinib hydrate, and filgotinib maleate, which were approved in 2020. Although HBV screening is recommended on the package insert prior to the use of JAK inhibitors, a domestic multicentre survey of first-time users has not been conducted.

This study aimed to survey the prevalence of HBsAg, HBcAb, and HBsAb screening prior to the initiation of drug therapy in patients with rheumatoid arthritis including new antirheumatic drugs, using a recent hospital-based administrative claims database.

## Methods and materials

### Data source

We employed the JMDC hospital-based administrative claims database (JMDC Inc., Tokyo, Japan) for this survey [24]. This database uses data collected from medical institutions in Japan, consisting of claims (for hospitalisation and outpatient treatment), diagnosis procedure combination (DPC) assessment forms, and clinical laboratory test values, and treatment details from April 2014 to August 2022 can be accessed; the number of medical institutions covered is approximately 600, consisting of DPC-eligible and DPC-ineligible hospitals. The total number of included patients is approximately 18 million. Diagnoses are registered based on the codes of International Classification of Diseases, Tenth Revision (ICD-10). Drug information is recorded based on the codes of Anatomical Therapeutic Chemical Classification System (ATC) from World Health Organization.

### Study design and population

**Fig. 1:**
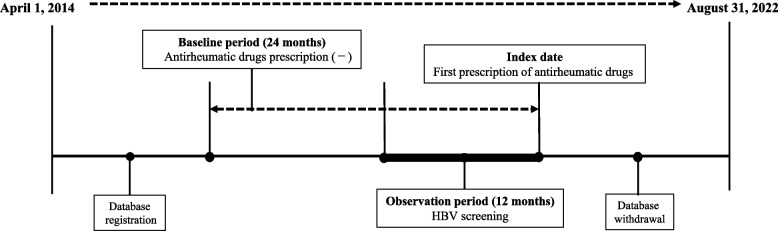
Scheme for selection of eligible patients. Cohort entry was defined as the date of registration with a health insurance provider or 1 April 2014, whichever occurred later. The index date was the first prescription date for antirheumatic drugs, and the observation period was 12 months before the index date. The baseline period was 24 months before the prescription, including a 12-month observation period before the prescription. HBV, Hepatitis B virus

This was a retrospective cross-sectional study. The scheme of patient selection is illustrated in Fig. 1. The inclusion criteria were rheumatoid arthritis and first prescription date of antirheumatic drugs between April 2016 and August 2022. The first prescription was defined as the prescription for the first antirheumatic drug prescribed to the patient. Specifically, if a patient received multiple types of antirheumatic drugs, we included only the first prescription. To identify the date of the first prescription (index date), we screened the data for 24 months, including a 12-month observation period, prior to the prescription for each patient. Patients with rheumatoid arthritis were diagnosed at baseline period (24 months before the index date). When prescribed more than once, only the date of the first dose was considered.

Exclusion criteria were as follows: (1) patients who were registered in the JMDC hospital-based administrative claims database within 24 months prior to the date of first prescription of antirheumatic drugs (to ensure a baseline period) and (2) patients with suspected rheumatoid arthritis (because of the possibility of being undiagnosed). Suspected diseases were detected from the "suspected flag" in the JMDC hospital-based administrative claims database.

### Data collection

Injuries owing to rheumatoid arthritis were identified using the ICD-10 codes (Additional file 1). Antirheumatic drugs were classified into conventional synthetic (cs) DMARDs, biological (b) DMARDs, and tsDMARDs based on the classification of efficacy by the European College of Rheumatology and JCR [6, 25]. bDMARDs were subdivided into cytotoxic T-lymphocyte antigen 4-immunoglobulin (CTLA4-Ig), TNF-α inhibitor, and anti-interleukin-6 receptor (IL-6R) therapies based on their pharmacological actions. tsDMARDs were classified as JAK inhibitors. The ATC codes used to identify each drug are listed in Additional file 2. HBV screening (HBsAg, HBsAb, and HBcAb) was performed using the practice codes (Additional file 3), and the date of screening during the baseline period was recorded. In addition, we collected data on the index date of patient backgrounds, including age, sex, and date of registration, in the JMDC Hospital-based administrative claims database, and date of withdrawal from the database. Information on the treatment was collected, including whether the prescription was inpatient or outpatient on the index date, the year and month of diagnosis of rheumatoid arthritis at the baseline period, prescribing department (“rheumatology/rheumatology or orthopaedics”), prescription history, date of prescription, number of days prescribed, and route of administration of antirheumatic drugs. In addition, data on prescribed corticosteroids (ATC code, H02A) during the baseline period were collected.

### Outcomes

The primary endpoint was the proportion of HBsAg, HBsAb, and HBcAb screening performed during the 12-month period preceding the index date of the first prescription of antirheumatic drugs. HBV screening test codes used in the current study were those employed in a previous Japanese database study [20]. Appropriate HBV screening was defined as screening performed for HBsAg, HBsAb, and HBcAb. In addition, we conducted a secondary analysis of the factors that prevented appropriate HBV screening.

### Data analysis

Patient background and treatment information on appropriate HBV screening were analysed using χ-squared or Fisher's exact probability test for categorical variables and Mann–Whitney's U test for continuous variables. Multivariate logistic regression analysis was performed to analyse the implementation of factors for performing HBV screening. The covariates included sex, inpatient or outpatient status, the prescribing department, classification by csDMARDs, bDMARDs, or tsDMARDs, concomitant use of steroids, and route of administration among the collected data. Data that showed significant differences in the univariate analysis and those that were clinically significant were included. Results with a *p*-value ≤0.05 were considered significant. All statistical analyses were performed using JMP Pro 17 software (SAS Institute, Inc., Cary, NC, USA.).

## Results

### HBV screening proportion and practice patterns

**Fig. 2:**
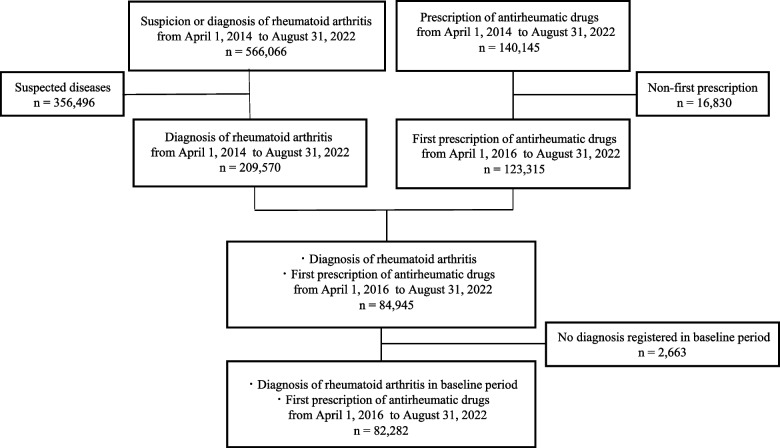
Flow chart of patient selection

**Fig. 3:**
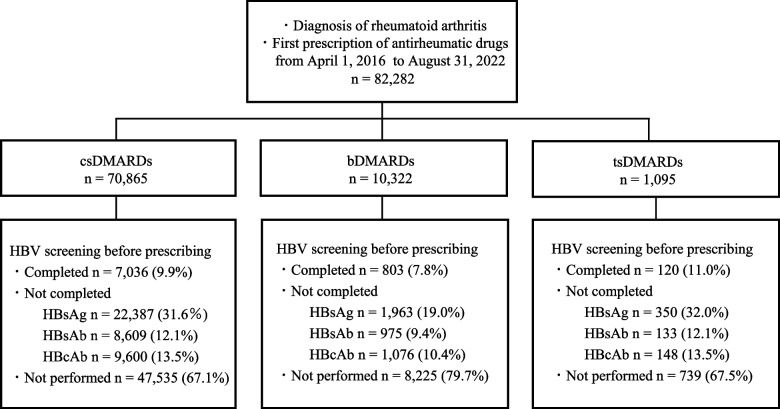
Proportion of HBV screening. This figure shows the proportion of HBV screenings performed among 82,282 patients with rheumatoid arthritis during the baseline period (24 months), with the first prescription date for antirheumatic drugs between April 2016 and August 2022. The proportion of tests performed is shown by the class of prescribed drugs. All HBV screening (HBsAg, HBsAb, and HBcAb) was performed. Not completed means at least one HBV screening was performed. HBV, Hepatitis B virus; HBsAg, hepatitis B surface antigen; HBsAb, hepatitis B surface antibody; HBcAb, hepatitis B core antibody; DMARDs, disease-modifying antirheumatic drugs; csDMARDs, conventional synthetic DMARDs; bDMARDs, biological DMARDs; tsDMARDs, targeted synthetic DMARDs

A total of 82,282 patients with rheumatoid arthritis were identified for the baseline period (24 months) and on the first prescription date for antirheumatic drugs between April 2016 and August 2022 (Fig. 2). Of the eligible patients, 9.7% (*n*=7,959) were tested for all HBV screening (HBsAg, HBsAb, and HbcAb) within 12 months prior to their initial prescription date. The HBsAg test was performed in 30.0% (*n*=24,700), HBsAb test in 11.8% (*n*=9,717), and HBcAb test in 13.1 (*n*=10,824) patients. The low proportion of antigen–antibody combination tests depended on the low proportion of antibody tests performed. By classification of drugs, 86.0% (*n*=70,865) patients on the index date administered csDMARDs, 12.5% (*n*=10,322) administered bDMARDs, and 1.3% (*n*=1,095) administered tsDMARDs on the index date (Fig. 3). All HBV screening was performed in 9.9, 7.8, and 11.0% of patients taking csDMARDs, bDMARDs, and tsDMARDs, respectively. The proportion of screening inpatients taking relatively new tsDMARDs was higher than that in patients taking other conventionally used DMARDs for rheumatoid arthritis. The screening proportion for each drug is shown in Table 1.

**Table 1:** Proportion of HBV screening performed for each drugs

	Total, n	Therapeutic Category (All^a)^, %)	All	HBsAg	HBsAb	HBcAb
**csDMARDs**	70,865					
Methotrexate	36,581	MTX (10.6)	3,893 (10.6)	1,121 (30.6)	4,736 (12.9)	5,252 (14.4)
Salazosulfapyridine	15,555	Other csDMARDs (9.2)	1,818 (11.7)	5,537 (35.6)	2,201 (14.1)	2,489 (16.0)
Leflunomide	187		5 (2.7)	37 (19.8)	9 (4.8)	8 (4.3)
Tacrolimus	7,731		612 (7.9)	2,111 (27.3)	760 (9.8)	792 (10.2)
Sodium aurothiomalate	98		3 (3.1)	8 (8.2)	3 (3.1)	3 (3.1)
Bucillamine	5,957		277 (4.6)	1,733 (29.1)	373 (6.3)	423 (7.1)
Iguratimod	4,542		419 (9.2)	1,698 (37.4)	516 (11.4)	623 (13.7)
Mizoribine	214		9 (4.2)	51 (23.8)	11 (5.1)	10 (4.7)
**bDMARDs**	10,322					
Abatacept	1,802	CTLA4-Ig (6.3)	114 (6.3)	321 (17.8)	147 (8.2)	177 (9.8)
Etanercept	1,961	TNF-α inhibitor (6.7)	109 (5.6)	348 (17.8)	135 (6.9)	148 (7.6)
Infliximab	670		28 (4.2)	70 (10.5)	38 (5.7)	36 (5.4)
Adalimumab	875		95 (10.9)	180 (20.6)	116 (13.3)	119 (13.6)
Certolizumab pegol	358		23 (6.4)	68 (19.0)	34 (9.5)	40 (11.2)
Golimumab	1,056		65 (6.2)	187 (17.7)	87 (8.2)	105 (9.9)
Tocilizumab	3,418	Anti-IL-6R therapy (10.3)	334 (9.8)	723 (21.2)	381 (11.2)	410 (12.0)
Sarilumab	182		35 (19.2)	66 (36.3)	37 (20.3)	41 (22.5)
**tsDMARDs**	1,095					
Tofacitinib citrate	543	Jak inhibitor (11.0)	36 (6.6)	102 (18.8)	40 (7.4)	49 (9.0)
Baricitinib	463		73 (15.8)	215 (46.4)	80 (17.3)	85 (18.4)
Upadacitinib hydrate	38		4 (10.5)	16 (42.1)	5 (13.2)	5 (13.2)
Filgotinib maleate	10		1 (10.0)	4 (40.0)	2 (20.0)	2 (20.0)
Peficitinib hydrobromide	41		6 (14.6)	13 (31.7)	6 (14.6)	7 (17.1)

*HBV* Hepatitis B virus, *HBsAg* Hepatitis B surface antigen, *HBsAb* Hepatitis B surface antibody, *HBcAb* hepatitis B core antibody, *DMARDs* Disease-modifying antirheumatic drugs, *csDMARDs* Conventional synthetic DMARDs, *bDMARDs* Biological DMARDs, *tsDMARDs* Targeted synthetic DMARDs, *MTX* methotrexate, *CTLA4-Ig* Cytotoxic T-lymphocyte antigen 4-immunoglobulin, *TNF* Tumour necrosis factor, *IL-6R* Interleukin-6 receptor. a) All; HBV screening (HBsAg, HBsAb, and HBcAb) was performed

### Factors associated with HBV screening

**Fig. 4:**
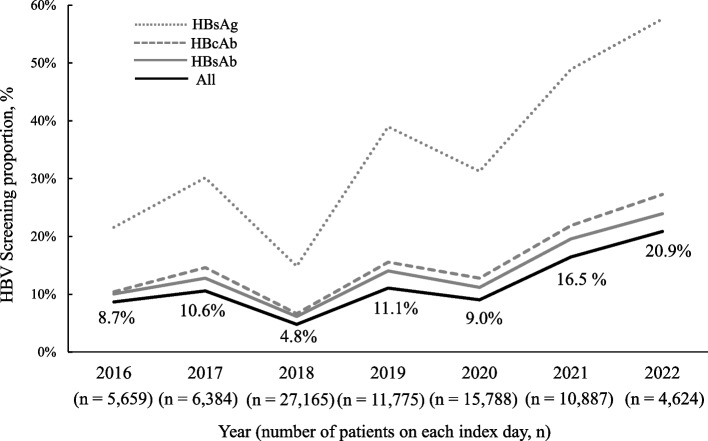
HBV screening (HBsAg, HBsAb, and HBcAb) proportion over time. This figure shows the proportion of HBV screening (HBsAg, HBsAb, and HBcAb) over the study period. The graph shows the proportion of patients who underwent the test on each index date. HBV, Hepatitis B virus; HBsAg, hepatitis B surface antigen; HBsAb, hepatitis B surface antibody; HBcAb, hepatitis B core antibody

The characteristics of patients who underwent all HBV screening tests and those without all HBV screening are shown in Table 2. There were no missing data in the collected items. In statistical analysis, males, patients with an initial outpatient prescription, and patients receiving concomitant steroids had all HBV screening performed before the date of the initial prescription. Multivariate logistic regression analysis including these variables showed that males, the first prescription as an outpatient, the prescribing department of rheumatology or orthopaedics, and patients administering steroids were involved in the implementation of tests (Table 3). Figure 4 compares the implementation of all HBV screenings by the index date year. The proportion of screenings increased after 2018.

**Table 2:** Comparison of characteristics between patients with completed and uncompleted HBV screening

	Screening completed	Screening uncompleted	*p*-value
N	7,959	74,323	
Age (years), Median (IQR)	69 (57–77)	70 (60–78)	
< 20	47 (0.6)	283 (0.4)	
20–39	466 (5.9)	3,120 (4.2)	
40–59	1,852 (23.3)	14,701 (19.8)	
60–79	4,150 (52.1)	40,070 (53.9)	
≥80	1,444 (18.1)	16,149 (21.7)	
Male, n (%)	2,474 (31.1)	19,226 (25.9)	<.0001*
Hospitalisation, n (%)	1,053 (13.2)	12,731 (17.1)	<.0001*
Prescribing department of rheumatology or orthopedics, n (%)	3,996 (50.2)	36,192 (48.7)	<.0001*
csDMARDs, n (%)	7,036 (88.4)	63,829 (85.9)	<.0001*
bDMARDs, n (%)	803 (10.1)	9,519 (12.8)	0.0103*
tsDMARDs, n (%)	120 (1.5)	975 (1.3)	0.1472
Steroid, n (%)	4,170 (52.4)	32,326 (43.5)	<.0001*
Injection, n (%)	820 (10.3)	9,625 (13.0)	<.0001*

*DMARDs* Disease-modifying antirheumatic drugs, *csDMARDs* Conventional synthetic DMARDs, *bDMARDs* Biological DMARDs, *tsDMARDs* Targeted synthetic DMARDs, *IQR* Interquartile range, *95% CI* 95% confidence interval. Odds ratios were used to compare the onset of anaphylaxis. *Results with *p*-values ≤ 0.05 were considered statistically significant

**Table 3:** Factors affecting HBV screening identified by multivariate logistic regression analysis

Characteristics	Odds ratio	95% CI	*p*-value
Male	1.26	1.21-1.33	<.0001*
Hospitalisation	0.70	0.65-0.75	<.0001*
Hospital department	1.05	1.01-1.10	0.0267*
csDMARDs	0.88	0.73-1.07	0.1950
bDMARDs	0.46	0.27-0.80	0.0048*
tsDMARDs (Reference)	1.00	-	-
Steroid	1.45	1.38-1.52	<.0001*
Injection	1.53	0.91-2.55	0.1064

^*^Results with *p*-values ≤ 0.05 were considered statistically significant. *HBV* Hepatitis B virus, *DMARDs* disease-modifying antirheumatic drugs, *csDMARDs* Conventional synthetic DMARDs, *bDMARDs* biological DMARDs, *tsDMARDs* Targeted synthetic DMARDs

## Discussion

The current study investigated the proportion of HBV screening in patients with rheumatoid arthritis prescribed antirheumatic drugs. No studies have been conducted using large Japanese medical information databases since 2014. The proportion of patients underwent tests for HBsAg, HBsAb, and HBcAb was only 9.7%, which was similar to the insufficient proportion identified in a previous survey [20]. It should be noted that the proportion of HBV screening (HBsAg, HBsAb, and HBcAb) in the most recent two-year period (2021–2022) had approximately doubled compared with the previous years (2016–2020).

Japanese guidelines recommend a combination of antigen and antibody testing and HBV screening prior to the initiation of immunosuppressive therapy, including chemotherapy and antirheumatic drugs, which are covered by insurance. However, the test for HBsAg is the most common, and the proportion of patients who tested for antigens and antibodies is low. In the US, the Centers for Disease Control and Prevention (CDC), American Gastroenterological Association (AGA), and American Association for the Study of Liver Diseases (AASLD) recommend testing for both antigens and antibodies, as in Japan. Specifically, in the USA, 43.4% of patients underwent a combination test [18]. In contrast, in Taiwan, the testing of both antigens and antibodies both is recommended; however, most tests have been performed for HBsAg only, and a combination test was only performed in 16.3% of patients [18]. In Taiwan, prophylactic administration of antiviral drugs is not covered by insurance for patients previously infected with HBV, which may be a reason for the low proportion of antibody testing [18]. In Japan, most of the tests have been performed for HBsAg only, despite the fact that the prophylactic treatment of previously infected patients with HBV-DNA detected above a certain amount is covered by insurance.

The proportion of patients with rheumatoid arthritis who were tested before immunosuppressive therapy was low, but the proportion of Japanese patients undergoing cancer chemotherapy tested for HBV antigen and antibodies was 41.3% in a 2014–2015 database [26]. The incidence of reactivation after chemotherapy was reportedly 20–50% in HBsAg-positive patients and 0.3–9.0% in previously infected patients (HBsAg negative/HBsAb or HBcAb positive) [27–29]. Despite a similar risk of HBV reactivation, patients with rheumatoid arthritis are undertested for HBV antigens and antibodies at a proportion of 9.7% and may be overlooked when compared with that in patients with cancer. Considering that rheumatoid arthritis is a chronic disease frequently treated with combinations of immunosuppressive drugs over a prolonged period, this is an important issue and needs to be addressed.

In our study, logistic regression analysis revealed that male patients, initial outpatient prescription, the prescribing department being rheumatology/collagen disease, and concomitant use of steroids were associated with HBV screening. Male patients and patients with concomitant use of steroids have been reported to increase the proportion of HBV screening [18], which is consistent with the results of the present study. In Japan, systemic administration of steroids for more than 2 weeks has been recognised as a risk factor for HBV reactivation, even if administered alone [30]. Although the history of hospitalisation affects the proportion of tests performed [18], the fact that the initial prescription is given in an outpatient clinic did not seem to substantially impact the proportion of tests performed. Comparing these results with previous reports was challenging because we had to determine whether the patients were treated as outpatients or inpatients at the time of their first prescription. Moreover, the fact that the prescribing departments were rheumatology and orthopaedics, which treat many patients with rheumatoid arthritis, affected the proportion of tests performed. A possible factor is that the JCR has issued an alert [5] regarding HBV reactivation, which is also described in the guidelines for treating rheumatoid arthritis in Japan [31]. By classification, patients prescribed tsDMARDs were more frequently tested for HBsAg, HBsAb, and HBcAb than those prescribed other drugs. tsDMARDs have been approved for use in patients who show an insufficient response to existing antirheumatic drugs. Although the actual reason is unknown, long-term safety has not been fully established, which may have an impact on the alerts regarding HBV screening tests in response to the guidelines. In addition, the differences between injectable and oral drugs did not affect the proportion of tests performed in the current study because most of the injectable drugs were subcutaneous formulations, and all tsDMARDs, which were associated with a high proportion of tests performed on patients, were oral drugs.

In the current study, although the proportion of HBV screening among patients with rheumatoid arthritis remained low, it has been rising since 2018. A survey on the prescribing trend of antirheumatic drugs in Japan [32] has revealed that several csDMARDs and approximately 20% of bDMARDs are prescribed as the first-line treatments; therefore, we consider that the lack of HBV screening for patients starting immunosuppressive therapy for the first time is most problematic. Among bDMARDs, IL-6 inhibitors that have stronger immunosuppressive effects than anti-inflammatory effects [33], had a higher screening proportion than those of TNF-α inhibitors. Additionally, the proportion of HBV screening at the initial prescription of tsDMARDs, a new class of antirheumatic drugs, was higher than that of current antirheumatic drugs (i.e. csDMARDs and bDMARDs); however, adequate screening was not performed. The usage of new biological agents for patients with rheumatoid arthritis is increasing, along with the number of immunosuppressive agents related to HBV reactivation. Immunosuppressive therapy for treating rheumatoid arthritis is used in several patients and administered over the long term. Furthermore, the proportion of patients tested for HBV before chemotherapy was much lower than that before immunosuppressive therapy, which needs to be addressed immediately to improve the proportion of patients with rheumatoid arthritis tested for HBV before immunosuppressive therapy.

As a solution, simplifying the process of test orders and education have increased the proportion of HBV screening [23], and the introduction of an automated decision support system within the electronic medical record system has resulted in more efficient HBV screening [34]. This suggests improvements in the process and system of educational intervention and test ordering for healthcare professionals.

Our study has several limitations. First, ascertaining all medical facilities attended by the patients was difficult owing to the characteristics of the database used in this study. The JMDC hospital-based administrative claims database was unable to identify whether the same individual had visited multiple hospitals. Therefore, there may be a history of previous HBV or RA treatment, or an HBV screening test may have been performed but not recorded in the database. Second, the accuracy of disease name as rheumatoid arthritis was not validated. Therefore, we used a previously reported definition of rheumatoid arthritis [20].

## Conclusion

We found that only 9.7% of patients were tested for HBV screening (HBsAg, HBsAb and HBcAb) prior to the first prescription of antirheumatic drugs from 2016 to 2022 in Japan, although this proportion has been on the rise since 2018. The proportion of patients receiving tsDMARDs, a new class of drugs, was also similar. With the increasing use of new immunosuppressive and antirheumatic drugs, including biological agents, healthcare providers should remain aware of the risk of HBV reactivation and conduct appropriate screening.

### Supplementary Information

**Supplementary Material 1.** 

**Supplementary Material 2.** 

**Supplementary Material 3.** 

## Data Availability

All data supporting the conclusions are included in the article.
