# Supplementary material for: A cross-sectional survey of hepatitis B virus screening in patients who received immunosuppressive therapy for rheumatoid arthritis in Japan
Source: J Pharm Health Care Sci. 2024 Apr 18;10:18. doi: 10.1186/s40780-024-00339-9 (PMC11025209; doi:10.1186/s40780-024-00339-9)
Supplement: Supplementary file 1 — Supplementary Material 1. [file 40780_2024_339_MOESM1_ESM.pdf]

Additional file 1. The list of encoded diseases related to rheumatoid arthritis.

Code*	Disease names
7143002	Juvenile idiopathic arthritis
7148003	Malignant rheumatoid arthritis
8842100	Rheumatic myopathy
8842101	Rheumatic carditis
8842102	Rheumatic myocarditis
8842103	Rheumatic pericarditis
8842104	Seropositive rheumatoid arthritis
8842105	Seronegative rheumatoid arthritis
8842106	Rheumatoid arthritis
8842136	Rheumatoid arthritis: Wrist
8842137	Rheumatoid arthritis: Finger joint
8842138	Rheumatoid arthritis: Knee joint
8842139	Rheumatoid arthritis: Foot joint
8842140	Rheumatoid arthritis: Coxa
8842141	Rheumatoid arthritis: Interphalangeal joints of foot
8842152	Rheumatoid arthritis: Glenohumeral joint
8842153	Rheumatoid arthritis: Elbow joint
8844635	Rheumatoid arthritis: Thoracic spine
8844636	Rheumatoid arthritis: Cervical spine
8844637	Rheumatoid arthritis: Spine
8844638	Rheumatoid arthritis: Lumbar spine
8846107	Rheumatoid arthritis: Temporomandibular joint
8847737	Rheumatic interstitial pneumonia

*Code for insurance claims provided by the Health Insurance Claims Review & Reimbursement Services.
