# Supplementary material for: A cross-sectional survey of hepatitis B virus screening in patients who received immunosuppressive therapy for rheumatoid arthritis in Japan
Source: J Pharm Health Care Sci. 2024 Apr 18;10:18. doi: 10.1186/s40780-024-00339-9 (PMC11025209; doi:10.1186/s40780-024-00339-9)
Supplement: Supplementary file 2 — Supplementary Material 2. [file 40780_2024_339_MOESM2_ESM.pdf]

Additional file 2. The list of encoded drugs related to antirheumatic drugs

ATC code	Drug names
csDMARDs	
A07EC01	Salazosulfapyridine
L04AA13	leflunomide
L04AD02	Tacrolimus hydrate
L04AX03	Methotrexate
M01CB01	Sodium aurothiomalate
M01CC02	Bucillamine
bDMARDs	
L04AA24	Abatacept (genetical recombination)
L04AB01	Etanercept (genetical recombination)
L04AB02	Infliximab (genetical recombination)
L04AB04	Adalimumab (genetical recombination)
L04AB05	Certolizumab pegol (genetical recombination)
L04AB06	Golimumab (genetical recombination)
L04AC07	Tocilizumab (genetical recombination)
L04AC14	Sarilumab (genetical recombination)
tsDMARDs	
L04AA29	Tofacitinib citrate
L04AA37	Baricitinib
L04AA44	Upadacitinib hydrate
L04AA45	Filgotinib maleate
L04AA49	Peficitinib hydrobromide

Code*	Drug names
csDMARDs	
622184501	Iguratimod
622882301	
621993201	Mizoribine
621993301	

ATC, the Anatomical Therapeutic Chemical Classification System; DMARDs, disease-modifying antirheumatic drugs; csDMARDs, conventional synthetic DMARDs; bDMARDs, biological DMARDs; tsDMARDs, targeted synthetic DMARDs. *Code for insurance claims provided by the Health Insurance Claims Review & Reimbursement

Services (ATC code was not assigned).
