# Supplementary material for: A cross-sectional survey of hepatitis B virus screening in patients who received immunosuppressive therapy for rheumatoid arthritis in Japan
Source: J Pharm Health Care Sci. 2024 Apr 18;10:18. doi: 10.1186/s40780-024-00339-9 (PMC11025209; doi:10.1186/s40780-024-00339-9)
Supplement: Supplementary file 3 — Supplementary Material 3. [file 40780_2024_339_MOESM3_ESM.pdf]

Additional file 3. The list of encoded laboratory tests for hepatitis B [1]

Code*	Laboratory tests
160046810	HB surface antigen qualitative/semi-quantitative
160047410	HB surface antibody semi-quantitative
160049210	HB surface antigen
160049510	HB surface antibody
160120710	HB core antibody semi-quantitative/quantitative
160195410	HB surface antibody qualitative

HB, hepatitis B. *Code for insurance claims provided by the Health Insurance Claims Review & Reimbursement Services.

[1] Fujita M, Sugiyama M, Sato Y, et al. Hepatitis B virus reactivation in patients with rheumatoid arthritis: Analysis of the National Database of Japan. J Viral Hepat. 2018;25:1312-1320. doi: 10.1111/jvh.12933.
